# Predicted cardiac and second cancer risks for patients undergoing VMAT for mediastinal Hodgkin lymphoma

**DOI:** 10.1007/s12094-022-03034-z

**Published:** 2022-12-31

**Authors:** Orla A. Houlihan, Georgios Ntentas, David J. Cutter, Patricia Daly, Charles Gillham, Orla McArdle, Frances K. Duane

**Affiliations:** 1 St Luke’s Radiation Oncology Network, Dublin, Ireland; 2 Nuffield Department of Population Health, University of Oxford, Oxford, UK; 3 Department of Medical Physics, Guy’s and St Thomas’ NHS Foundation Trust, London, UK; 4 School of Biomedical Engineering and Imaging Sciences, King’s College London, London, UK; 5 Oxford Cancer Centre, Oxford University Hospitals NHS Foundation Trust, Oxford, UK; 6 Trinity St James’s Cancer Institute, St. James’s Hospital, Dublin, Ireland; 7 School of Medicine, Trinity College Dublin, Dublin, Ireland; 8 Present Address: Patrick G. Johnston Centre for Cancer Research, Queen’s University Belfast, 97 Lisburn Road, Belfast, BT9 7AE UK

**Keywords:** Mediastinal lymphoma, Chemotherapy, Radiotherapy, Cardiovascular disease, Second cancer

## Abstract

**Background and purpose:**

To predict treatment-related cardiovascular disease (CVD) and second cancer 30-year absolute mortality risks (AMR_30_) for patients with mediastinal Hodgkin lymphoma in a large multicentre radiation oncology network in Ireland.

**Material and methods:**

This study includes consecutive patients treated for mediastinal lymphoma using chemotherapy and involved site radiotherapy (RT) 2016–2019. Radiation doses to heart, left ventricle, cardiac valves, lungs, oesophagus, carotid arteries and female breasts were calculated. Individual CVD and second cancer AMR_30_ were predicted using Irish background population rates and dose–response relationships.

**Results:**

Forty-four patients with Hodgkin lymphoma were identified, 23 females, median age 28 years. Ninety-eight percent received anthracycline, 80% received 4–6 cycles ABVD. Volumetric modulated arc therapy (VMAT) ± deep inspiration breath hold (DIBH) was delivered, median total prescribed dose 30 Gy. Average mean heart dose 9.8 Gy (range 0.2–23.8 Gy). Excess treatment-related mean AMR_30_ from CVD was 2.18% (0.79, 0.90, 0.01, 0.13 and 0.35% for coronary disease, heart failure, valvular disease, stroke and other cardiac diseases), 1.07% due to chemotherapy and a further 1.11% from RT. Excess mean AMR_30_ for second cancers following RT were: lung cancer 2.20%, breast cancer in females 0.34%, and oesophageal cancer 0.28%.

**Conclusion:**

For patients with mediastinal lymphoma excess mortality risks from CVD and second cancers remain clinically significant despite contemporary chemotherapy and photon-RT. Efforts to reduce the toxicity of combined modality treatment, for example, using DIBH, reduced margins and advanced RT, e.g. proton beam therapy, should be continued to further reduce potentially fatal treatment effects.

**Supplementary Information:**

The online version contains supplementary material available at 10.1007/s12094-022-03034-z.

## Introduction

The majority of patients treated for lymphoma do not die from the disease [1] and are at risk of long-term treatment-related morbidity and mortality [2]. For early stage Hodgkin lymphoma (HL), 5 year overall survival rates are in excess of 90% with the use of combined modality therapy [1]. Research has focused on identifying the minimum therapeutic intervention that will maintain outcomes and minimise survivor exposure to late toxicity of treatment [1, 3].

Sequential clinical trials replaced historic extended radiation fields with smaller fields and reduced target volumes including involved field [3], involved site [4] and involved node [1] treatment approaches. Randomised data supported prescribed dose reductions in early favourable stage disease found to be adequately treated with 20 Gy instead of 30 Gy [5]. Optimal photon radiotherapy (RT) planning techniques may further improve the therapeutic index for patients requiring treatment. Deep inspiration breath hold (DIBH) has been shown to reduce heart and lung doses [6, 7]. Volumetric modulated arc therapy (VMAT) planning techniques used in combination with DIBH are associated with reduced heart, lung and breast doses [8–10]. Proton beam therapy (PBT) may provide additional dosimetric advantages in the treatment of patients with mediastinal lymphoma and may reduce the risk of late effects [11].

However, patients requiring RT to the mediastinum remain at an increased risk of late adverse effects. These include cardiovascular disease (CVD) [12] and second malignancies such as breast, lung and oesophageal cancer [2], all of which cause morbidity and potentially excess mortality.

This study aims first to present the radiation doses received by individual patients who have undergone photon RT for mediastinal HL in a large multicentre radiation oncology network in Ireland, and second, to predict 30-year absolute mortality risks (AMR_30_) for CVD and second primary cancers. These data are intended to inform the need for ongoing treatment optimisation, to aid the consent process for future patients and to guide subsequent surveillance for lymphoma survivors.

## Materials and methods

### Patient population

All patients who underwent RT for mediastinal lymphoma in a multicentre radiation oncology network between January 2016 and September 2019 inclusive were identified and their medical records were reviewed. There were no exclusion criteria regarding treatment of additional sites to the mediastinum, for example, axilla and neck. Patients who could tolerate DIBH were planned in both free breathing (FB) and DIBH and the optimal plan with greatest tumour coverage and lowest dose to organs at risk (OARs) was selected for treatment. Baseline characteristics were recorded including age, sex, smoking status, histological diagnosis, Ann Arbor stage, indication for treatment, RT dose fractionation schedule, use of DIBH and details of chemotherapy received. This study was approved by the St Luke’s Radiation Oncology Network ethics committee.

### CT simulation and contouring

During RT CT simulation, patients were positioned with their arms by their sides and immobilised with the aid of a thermoplastic mask. The clinical target volume (CTV) for each patient was delineated based on guidelines for involved site RT [13]. During contouring, the patient’s pre-chemotherapy and interim or post-chemotherapy PET/CT was displayed on an adjacent monitor to aid delineation. The OARs of interest were contoured; whole heart, left ventricle, all four cardiac valves, lungs, female breasts, oesophagus and common carotid arteries. The left ventricle, cardiac valves and common carotid arteries were retrospectively contoured for the purposes of risk calculation for this study and were not included in the original planning process. Contour definitions and guides were derived from published atlases and information from anatomy and cardiac imaging textbooks and IMAIOS e-anatomy (Table S1) [14–17]. CTV to planning target volume (PTV) margins of 5–15 mm were at the discretion of the treating clinician taking individual disease distribution into consideration.

### Planning and dosimetric data collection

All patients were planned in the Eclipse planning system (Versions 13.6, 15.1 and 15.6), Varian Medical Systems, using VMAT. The standard approach for early stage favourable HL is to treat with 20 Gy in 10 fractions [5]. Patients with early stage unfavourable HL were treated with 30 Gy in 15 fractions. All cases are discussed at a dedicated lymphoma MDT and our overall approach is to include radiotherapy in the treatment plan only when potential late effects are considered acceptable—for example, radiotherapy for early stage disease may be omitted in the case of a young woman for whom breast tissue will be included in the treatment field. Doses higher than 30 Gy were used in the setting of residual PET/CT-positive disease. In these cases a total dose of 36–40 Gy is recommended [13]. Individualised VMAT plans were generated for each patient. A generic starting point of two full arcs was further developed with the addition of partial arcs and modification of existing full arcs to achieve maximal coverage and conformality while minimising the dose to OARs [18]. In our network, efforts are made to reduce the dose to all OARs to as low as reasonably achievable (ALARA). If possible, mean heart and lung doses were maintained < 5 Gy and 8 Gy respectively. In the setting of bulky mediastinal disease or residual PET/CT-positive disease in the mediastinum, higher lung and heart doses were accepted, with maximum accepted limits of mean lung dose (MLD) < 20 Gy, the volume of lung receiving ≥ 20 Gy (V20) less than 30% and mean heart dose (MHD) < 20 Gy. Mean breast dose was maintained < 4 Gy where possible. Each plan was optimised to achieve optimal conformity and PTV coverage of at least 98% of the PTV receiving at least 95% of the prescription dose. The mean doses to all contoured OARs were extracted, as well as PTV volume.

### Late effects risk prediction

The treatment-related AMR_30_ for CVD and second primary cancers were predicted for all patients in this study. The risk prediction methods are described in detail in two previous studies [11, 19]. In brief, for each patient, the background cumulative AMR_30_, in the absence of any lymphoma-related or treatment-related risks, was estimated for each disease of interest using mortality rates in the general Irish population and by taking into account the competing risk of death from other causes. These mortality rates were the most recent five year age- and sex-specific death rates from coronary heart disease (CHD), congestive heart failure (CHF), valvular heart disease (VHD), stroke and “other cardiac diseases” and from cancers of the lung, breast (females only) and oesophagus available from the World Health Organization (WHO) mortality database [20]. The radiation-related increase in the mortality rates was calculated using individual radiation doses combined with published dose–response relationships. The dose responses allow the relative risk from modern radiation techniques to be estimated even though they are based on historical cohorts who received on average, higher radiation doses to normal tissues when treated. Mean heart dose was used to estimate risk of CHD [21], mean left ventricular dose (MLVD) for risk of CHF [22], a weighted average of the mean doses to the aortic valve (AVMean), the mitral valve (MVMean) and the tricuspid valve (TVMean) for risk of VHD [23] and mean carotid artery doses (MCA) for risk of stroke [20, 24]. For CHF, separate calculations were carried out including the effect of anthracycline chemotherapy without RT as well as for the effect of combined modality treatment [22]. The sum of the mortality rates from CHD, CHF, VHD, stroke and “other cardiac diseases”, comprised the total CVD risk. Mean lung dose, mean breast dose (MBD) and mean oesophagus dose (MOD) were used to estimate risk from lung, breast and oesophageal cancers, respectively [24–29]. Smoking status could not be taken into account due to unknown smoking status for approximately half of patients and lack of separate population rates for smokers and never smokers in Ireland.

## Results

In total, 44 patients with mediastinal HL fulfilled the inclusion criteria for our study (21 male, 23 female). (Table 1). The median age at treatment was 28 years (range 17–72). The majority of patients (*n* = 27, 61%) were treated for early stage unfavourable disease. Two patients were treated for early stage favourable disease—both of these patients received 30 Gy in 15 fractions. In both cases they had a complete response documented on post-chemotherapy PET/CT but had persistent disease on interim PET/CT after 2 cycles of ABVD. All received chemotherapy, 98% (*n* = 43) received anthracycline regimens, 80% (*n* = 35) received 4–6 cycles ABVD. Median prescribed RT dose was 30 Gy (range 30–46 Gy). Forty-one percent (*n* = 18) were treated in DIBH.

**Table 1:** Patient, tumour and treatment characteristics

Baseline characteristic	Hodgkin lymphoma	
(a) Patient		
Age at treatment (years)	Median (range)	
Male	27 (17–68)	
Female	32 (21–72)	
Sex	*n*	*(%)*
Male	21	(48)
Female	23	(52)
Smoking status		
Current smoker	4	(7)
Ex-smoker	8	(16)
Non-smoker	13	(25)
Unknown	19	(52)
(b) Tumour		
Stage		
Early stage favourable	2	(5)
Early stage unfavourable	27	(61)
Refractory disease	8	(18)
Relapsed disease	6	(14)
Consolidation to site of initial bulk	1	(2)
(c) Treatment		
Radiotherapy technique		
DIBH VMAT	18	(41)
FB VMAT	26	(59)
Radiotherapy dose (Gy)		
30	34	(77)
36	2	(5)
40	7	(16)
46	1	(2)
Chemotherapy		
Yes	44	(100)
No	0	(0)
Number of cycles ABVD		
0	3	(7)
2	4	(9)
4	19	(43)
5	1	(2)
6	15	(34)
Unknown	2	(5)
Number of lines of chemotherapy		
1	29	(66)
2	9	(20)
3	6	(14)
Total number of patients	44	(100)

*ABVD* Adriamycin, Bleomycin, Vinblastine, Dacarbazine, *CTV* clinical target volume, *DIBH* deep inspiration breath hold, *FB* free breathing, *VMAT* volumetric modulated arc therapy

Average PTV volume was 1007.7 cc. Mean heart dose was 9.8 Gy (range 0.2–23.8 Gy). The aortic and pulmonary valves received higher doses of radiation than the mitral and tricuspid valves due to their more proximal location to the PTV. PTV volumes and mean doses delivered to OARs grouped by prescribed RT dose and sex are outlined in Table 2.

**Table 2:** Mean radiation doses to organs at risk from optimised photon-RT for patients with mediastinal Hodgkin Lymphoma in Ireland 2016–2019

				Mean organ at risk doses (Gy) (range)						
				Cardiac structures						
Prescribed (100%) dose	Mean PTV volume (cc) (range)	Sex	Patient number	Whole heart	Left ventricle	Pulmonary valve	Aortic valve	Mitral valve	Tricuspid valve	Four cardiac valves combined*
30 Gy	1017.5 (29.5–3186.9)	All	34	10.0 (0.2–23.8)	6.8 (0.1–23.4)	24.3 (0.3–29.8)	15.2 (0.2–29.6)	9.7 (0.2–24.2)	7.3 (0.1–24.1)	12.5 (0.2–26.0)
	1143.9 (29.9–3186.9)	Male	18	8.9 (0.2–23.8)	6.0 (0.1–23.4)	24.1 (0.3–29.8)	14.0 (0.2–29.6)	8.4 (0.2–22.7)	6.1 (0.1–22.0)	11.4 (0.2–26.0)
	875.4 (29.5–1561.5)	Female	16	11.4 (0.4–20.2)	7.7 (0.3–22.7)	24.4 (0.7–29.6)	16.5 (0.4–26.3)	11.0 (0.3–24.2)	8.7 (0.2–24.1)	13.9 (0.4–23.4)
36 Gy	1754.0	Male	1	11.3	6.7	33.9	20.4	6.5	7.9	14.3
	1732.5	Female	1	15.3	8.9	28.0	18.5	7.2	13.8	14.0
40 Gy	609.0 (352.6–865.3)	Male	2	10.2 (2.4–18.0)	3.8 (1.8–5.9)	36.3 (33.7–38.9)	21.7 (17.6–25.8)	6.5 (3.1–9.8)	13.2 (2.5–14.0)	15.4 (11.1–19.8)
	954.2 (556.7–1260.8)	Female	5	5.2 (1.4–8.3)	1.8 (0.5–3.6)	20.0 (5.3–31.1)	10.6 (2.5–18.0)	4.0 (0.8–10.9)	2.6 (0.5–6.4)	7.5 (1.7–14.4)
46 Gy	264.8	Female	1	16.7	8.8	17.0	15.8	12.3	18.6	14.7

				Other organs at risk							
Prescribed (100%) dose	Mean PTV volume (cc) (range)	Sex	Patient number	Carotid arteries			Breasts			Lungs	Oesophagus
				Left	Right	Both	Left	Right	Both		
30 Gy	1017.5 (29.5–3186.9)	All	34	26.1(10.1–30.5)	25.3 (14.0–30.2)	25.6 (12.5–30.1)	6.5 (0.2–14.7)	4.8 (0.3–10.6)	5.6 (0.2–12.2)	10.6 (2.0–16.9)	15.8 (6.8–30.0)
	1143.9 (29.9–3186.9)	Male	18	26.1 (10.1–30.5)	24.8 (14.0–30.0)	25.3 (12.5–30.1)				9.6 (2.0–16.9)	14.8 (6.8–22.2)
	875.4 (29.5–1561.5)	Female	16	26.0 (14.6–30.2)	25.9 (17.9–30.2)	25.8 (18.3–30.1)	6.5 (0.2–14.7)	4.8 (0.3–10.6)	5.6 (0.2–12.2)	11.6 (3.7–16.0)	16.9 (7.8–30.0)
36 Gy	1754.0	Male	1	36.6	36.3	36.4				13.3	22.1
	1732.5	Female	1	25.4	32.7	29.7	6.9	11.2	9.0	13.3	18.7
40 Gy	609.0 (352.6–865.3)	Male	2	8.7 (1.5–16.0)	11.6 (3.5–19.7)	10.4 (2.5–18.4)				9.2 (5.4–13.1)	8.8 (2.2–15.5)
	954.2 (556.7–1260.8)	Female	5	36.6 (33.2–40.2)	37.3 (34.0–40.2)	37.0 (33.7–40.2)	3.6 (2.4–4.3)	3.7 (1.6–5.0)	3.6 (2.0–4.5)	10.2 (7.6–13.0)	17.4 (13.2–21.5)
46 Gy	264.8	Female	1	0.4	0.6	0.5	8.6	15.9	12.3	9.9	6.5

*Combined valve dose = (0.553 × AVMean) + (0.368 × MVMean) + (0.079 × TVMean), i.e. a weighted average of the mean doses to the aortic valve (AVMean), the mitral valve (MVMean) and the tricuspid valve (TVMean) based on data from Cutter at el. [23]

*AV* aortic valve, *MV* mitral valve, *PTV* planning target volume, *TV* tricuspid valve

**Fig. 1:**
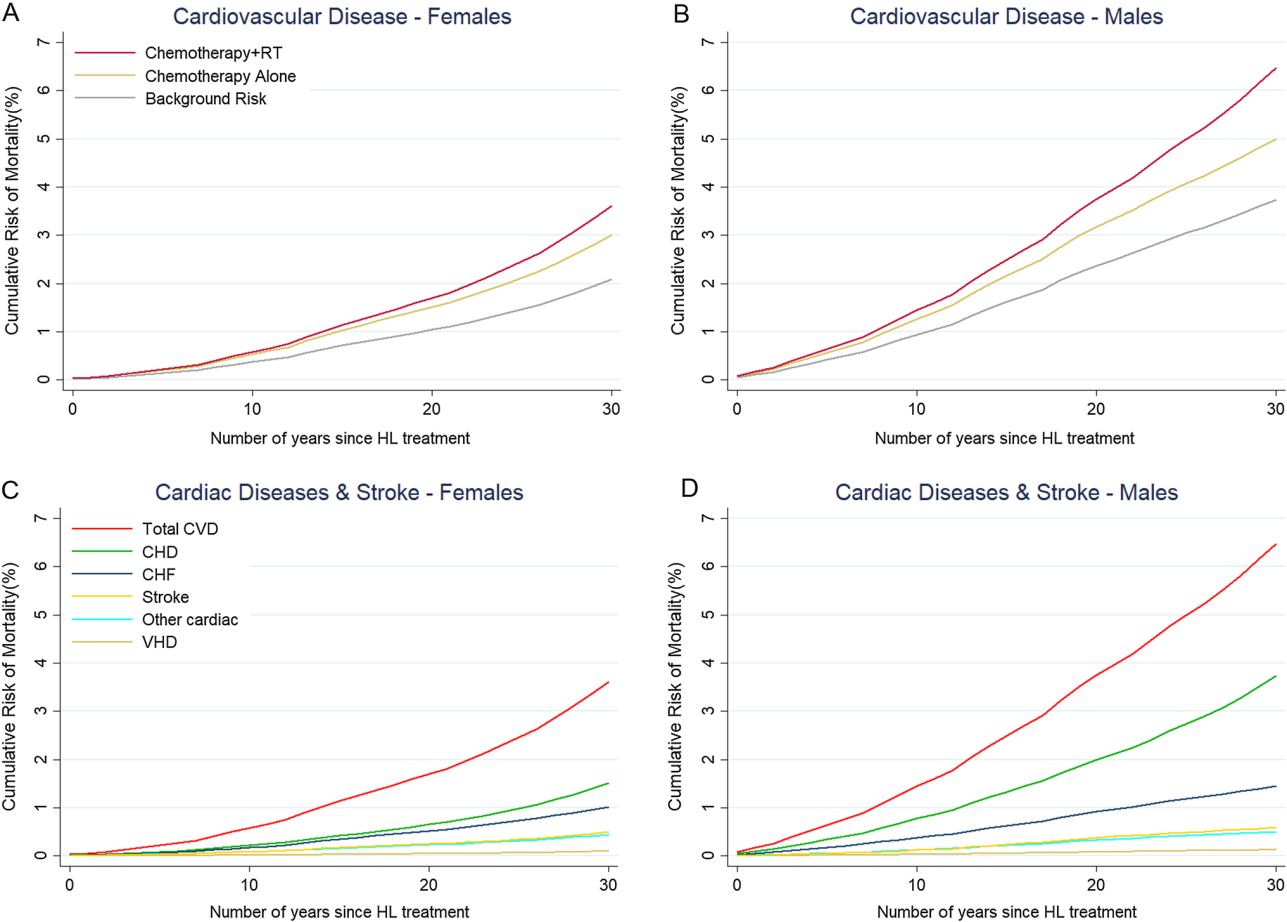
Estimated 30-year absolute mortality risks (AMR_30_) for cardiovascular disease for patients with Hodgkin Lymphoma in Ireland 2016–2019. **A** Average cumulative AMR_30_ for cardiovascular disease for females and **B** males including background mortality risks (grey lines), excess risks after chemotherapy (CT) (yellow lines), and excess risks after RT (red lines). **C** Average cumulative AMR_30_ for different types of cardiovascular disease for females and **D** males treated with chemotherapy and radiotherapy. For each patient, background AMR_30_ was estimated based on his/her age and sex at treatment. The chemotherapy-related AMR_30_ was estimated using the excess rate ratio from Van Nimwegen et al. [22] The radiotherapy-related AMR_30_ was estimated using the excess rate ratios or excess relative risks from various radiation dose–response relationships [21–24] combined with patient-specific radiation doses

The predicted background AMR_30_ from CVD was 2.88% and the excess AMR_30_ was 1.07% with chemotherapy and a further 1.11% with RT to a total of 5.06% (Table 3, Fig. 1). The chemotherapy-related excess AMR_30_ of CVD was 0.86% and 0.21% for CHF and other cardiac diseases, respectively. The radiation-related excess AMR_30_ of CVD was 0.79% for CHD, 0.04% for CHF, 0.01% for VHD, 0.13% for stroke, and 0.14% for other cardiac diseases. While the OARs of male patients did not receive greater radiation doses compared to the doses received by female patients, the predicted AMR_30_ for all CVD was greater in males due to their higher background risk, 3.74% versus 2.09% for females. (Table 2, Fig. 1).

**Table 3:** Estimated 30-year cumulative absolute mortality risks (AMR_30_) from CVD and second cancers following chemotherapy and radiotherapy for patients with mediastinal Hodgkin lymphoma treated in Ireland 2016–2019

	Mean AMR_30_ (%) (range)			
Sex	Background risk	Chemotherapy-related excess AMR_30_	Radiotherapy-related excess AMR_30_	Total AMR_30_
Cardiovascular disease				
Total CVD				
All	2.88 (0.15–14.65)	1.07 (0.12–6.01)	1.11 (0.12–3.46)	5.06 (0.42–22.26)
M	3.74 (0.34–14.65)	1.26 (0.19–4.94)	1.57 (0.12–3.47)	6.57 (0.65–22.26)
F	2.09 (0.15–14.62)	0.90 (0.12–6.01)	0.69 (0.13–1.35)	3.68 (0.42–21.62)
Coronary heart disease				
All	1.78 (0.06–9.14)	–	0.79 (0.07–3.19)	2.57 (0.16–11.02)
M	2.51 (0.21–9.14)	–	1.23 (0.07–3.19)	3.74 (0.31–11.02)
F	1.11 (0.07–7.02)	–	0.40 (0.07–0.91)	1.51 (0.16–7.14)
Congestive heart failure				
All	0.33 (0.09–1.68)	0.86 (0.09–4.43)	0.04 (0.00–0.13)	1.23 (0.13–6.13)
M	0.39 (0.05–1.50)	1.03 (0.14–3.96)	0.03 (0.00–0.12)	1.45 (0.19–5.49)
F	0.27 (0.04–1.68)	0.71 (0.09–4.44)	0.03 (0.00–0.13)	1.01 (0.13–6.12)
Valvular heart disease				
All	0.10 (0.00–0.72)	–	0.01 (0.00–0.05)	0.11 (0.00–0.72)
M	0.12 (0.01–0.53)	–	0.01 (0.00–0.05)	0.13 (0.01–0.58)
F	0.09 (0.00–0.72)	–	0.01 (0.00–0.05)	0.10 (0.00–0.72)
Stroke				
All	0.41 (0.01–3.35)	–	0.13 (0.00–0.85)	0.54 (0.02–4.20)
M	0.45 (0.01–2.34)	–	0.13 (0.00–0.58)	0.58 (0.02–2.91)
F	0.38 (0.02–3.35)	–	0.12 (0.00–0.86)	0.50 (0.04–4.20)
Other cardiac				
All	0.26 (0.03–1.84)	0.21 (0.03–1.56)	0.14 (0.02–0.61)	0.61 (0.07–4.02)
M	0.27 (0.06–1.14)	0.23 (0.05–0.99)	0.17 (0.03–0.53)	0.67 (0.14–2.67)
F	0.25(0.02–1.84)	0.19 (0.03–1.56)	0.13 (0.02–0.62)	0.57 (0.07–4.02)
Second cancer				
Breast				
F	1.00 (0.31–2.31)	–	0.34 (0.02–0.74)	1.34 (0.47–2.44)
Lung				
All	1.07 (0.04–4.16)	–	2.20 (0.60–8.79)	3.27 (0.10–12.96)
M	1.11 (0.04–4.16)	–	1.89 (0.06–8.79)	3.00 (0.10–12.95)
F	1.02 (0.09–3.45)	–	2.48 (0.27–6.86)	3.50 (0.35–9.40)
Oesophagus				
All	0.20 (0.01–0.99)	–	0.28 (0.01–1.37)	0.48 (0.02–2.37)
M	0.30 (0.02–0.99)	–	0.36 (0.01–1.37)	0.66 (0.03–2.37)
F	0.12 (0.01–0.47)	–	0.20 (0.01–0.75)	0.32 (0.02–1.21)

*F* female, *M* male

**Fig. 2:**
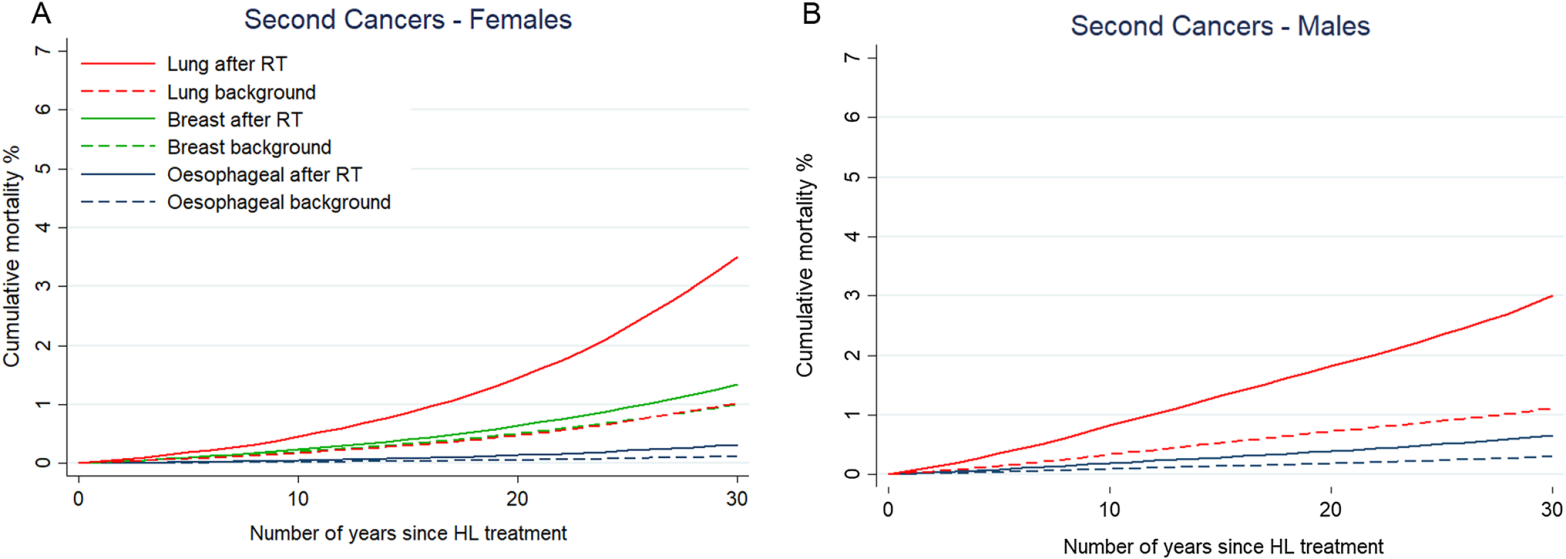
Estimated 30 year absolute mortality risks (AMR_30_) for second cancers for patients with Hodgkin Lymphoma in Ireland 2016–2019. **A** Average cumulative AMR_30_ for second cancers for females and **B** males including background mortality risks (dotted lines) for each cancer type and excess risks after radiotherapy (solid lines). For each patient, background AMR_30_ was estimated based on his/her age and sex at treatment. The radiotherapy-related AMR_30_ was estimated using the excess rate ratios or excess relative risks from various radiation dose–response relationships [24–29] combined with patient-specific radiation doses

The predicted background AMR_30_ from second cancers was: lung cancer 1.07%, breast cancer in females 1.00% and oesophageal cancer 0.20%. The excess mean AMR_30_ following RT was: lung cancer 2.20%, breast cancer in females 0.34%, and oesophageal cancer 0.28%, leading to a total AMR_30_ of 3.27, 1.34 and 0.48% from lung, breast and oesophageal cancer respectively. Among both male and female patients, the greatest additional risk was for lung cancer (1.89% for males, 2.48% for females; Table 3, Fig. 2). Since the background AMR_30_ from lung cancer was similar for male and female patients, the higher excess lung cancer risk in women was due to the higher lung doses.

## Discussion

This study provides a patient-specific prediction of treatment-related absolute mortality risk from mediastinal photon RT and chemotherapy in a cohort of HL survivors treated in a national oncology network in Ireland. Our findings show that risks are low but remain clinically significant. Efforts to reduce incidental radiation doses by introducing more advanced RT techniques and reducing target volumes by increasing use of DIBH should be continued. We also provide individualised risk estimates which can be used to inform future patients and increase their involvement in decision making regarding balancing risks and benefits from their treatment. Lastly, we demonstrate that expanding long-term surveillance may provide a more holistic treatment approach to patients. For example, guidelines are in place for breast cancer screening but our data suggest the role of lung cancer screening may need to be considered.

For survivors treated with 30 Gy to the mediastinum, the radiation doses in our cohort are similar to those reported for modern photon RT in a recent review [30]. Mean lung, heart and oesophagus doses were 10.6 Gy, 10.0 Gy and 15.8 Gy, respectively. Mean breast dose in female patients was 5.6 Gy (Table 2).

Excess mean AMR_30_ of CVD following chemotherapy and RT was 2.18%, with 1.07% attributed to chemotherapy and 1.11% to RT. Our AMR_30_ were similar to those predicted in a recent study in a different population of early stage HL patients [19]. RT was also associated with excess AMR_30_ for second cancers (lung cancer 2.20%, breast cancer in females 0.34%, and oesophageal cancer 0.28%). The total risk of lung cancer mortality more than tripled for women (from 1.02 to 3.50%) and more than doubled for men (from 1.11% to 3.00%). This is due to the higher mean lung doses recorded for women in this study as both men and women had on average similar background AMR_30_ in our study. The predicted risk of breast and oesophageal cancer mortality was generally low and the RT-related increase of breast AMR_30_ in women was also low (0.34%). For oesophageal cancer it was tripled in women after RT (from 0.12 to 0.32%), with a smaller relative, but larger absolute, increase seen for men (from 0.30 to 0.66%). While the overall absolute mortality risks are low for patients with HL, these fatal effects remain clinically significant. These RT-related risks vary widely among patients as shown in Table 3, with a range of 0.12 to 3.46% for excess AMR_30_ of CVD and 0.60 to 8.79% for excess AMR_30_ of lung cancer mortality and 0.01 to 1.37% for oesophageal cancer mortality.

Approaches to reduce the risk of late cardiovascular and second cancer risks for patients with HL include the adoption of advanced radiotherapy techniques such as DIBH. While DIBH has the potential to reduce radiation dose to OARs in mediastinal lymphoma for some patients, it is not always superior to FB techniques and is not always tolerable by patients [18]. In our network, despite dual planning for all patients who could tolerate DIBH, these plans were superior to FB plans for only 41% of the overall cohort. In our network, however, the same PTV margins are applied for both DIBH and FB plans. Enhanced image guidance techniques can facilitate the use of tighter PTV margins with DIBH, which likely would reduce the dose to OARs further and provide added benefit [7, 10]. Tighter margins combined with state of the art radiotherapy techniques such as butterfly VMAT [9, 10] or full-arc butterfly VMAT [31] could provide further OAR dose and risk reductions.

Alternatively, PBT may also offer an advantage compared to photon RT, however, it is not widely available yet for patients with HL due to higher cost and limited numbers of PBT centres, with no centre currently available in Ireland. Identifying patients who will derive greatest benefit from PBT would be beneficial but it remains a challenge in clinical practice. The International Lymphoma Radiation Oncology Group guidelines state that patients who could “greatly benefit” from PBT include those with mediastinal disease that extends below the origin of the left main coronary artery (LMCA), those for whom breast dose is a concern and heavily pre-treated patients at higher risk of radiation-induced toxicity [32]. Another recent study showed that when CTV overlapped longitudinally with the heart by ≥ 40%, PBT reduced mean heart dose by 3.2 Gy (19% relative decrease), left ventricular dose by 5.6 Gy (50% relative decrease) and valvular doses by 5.1 Gy (24% relative decrease). These measures resulted in a reduction in total CVD AMR_30_ in the study among patients in Western Europe by 0.3% from 3.8% to 3.5% [11]. For patients with axillary involvement, PBT reduced mean lung dose by 2.8 Gy (29% relative decrease) and lung cancer AMR_30_ among patients in Western Europe by 0.5% from 2.7 to 2.2% [11]. Based on the above findings, we calculated the percentage of patients in our cohort falling within these subgroups (Table S2). Forty-one percent (18/44) of patients had a CTV which overlapped longitudinally with the heart by ≥ 40% [11], 70% (31/44) had mediastinal disease which extended below the origin of the LMCA [32] and 52% (23/44) patients had disease which extended below the 7th thoracic vertebra [33]. These patients could have potentially had reduced risk of radiation-related AMR_30_ from CVD if treated with PBT. In addition, the 16 patients (36%) who had axillary disease treated could have potentially had reduced risk of radiation-related AMR_30_ from second lung and breast cancer (females) [11, 32]. However, only dual planning with both photon RT and PBT within our cohort could provide a more individualised estimate as the range of absolute AMR_30_ benefit is wide amongst patients based on previously published results [22].

A strength of our study is that we used an individualised risk prediction approach which takes into account individual OAR doses from radiotherapy CT-planning scans detailing anatomy and 3D dose distributions for each patient combined with the best available epidemiological evidence regarding the magnitude of the long-term risks of radiation and anthracycline chemotherapy in HL survivors to predict AMR_30_, as well as up to date age and sex-specific background Irish risks as recently published by the WHO. We recognise that the dose–response relationships used as the basis for our methodology are currently based on data from patients treated with historical techniques but these relationships remain the best epidemiological evidence available that provide dose-response relationships between radiation dose and risk of cardiovascular disease and second cancers. The nature of late manifestation of such effects requires a long patient follow up and the results of such studies will always be behind the fast evolving RT technologies. These dose-response relationships provide the relative risks (recognised from these historical treatments) of late effects versus radiation doses to the relevant OARs which can be used to estimate absolute risks for contemporary cohorts. The majority of cardiovascular risk dose–response relationships used in our study have been recently externally validated, giving us more confidence in their use [34]. When newer studies are published that adequately define dose–response relationships within more modern cohorts, updates to the risk estimation models will be important. We also included all patients treated with mediastinal RT in a RT network delivering just over 40% of all RT courses nationally—this likely is a reasonable national representation of RT dosimetry for this cohort. Although only 44 patients were identified this identifies a substantial ‘real world’ cohort who actually received these treatments rather than a more constrained planning study.

Our study has several limitations. We have included a population of varying clinical demographics, disease stages and RT doses, which, while reflective of daily clinical practice, makes it difficult to draw conclusions regarding more selected groups of patients. The retrospective nature of this study, specifically the retrospective contouring of cardiac substructures and carotid arteries, limits the interpretation of some of the risks that were predicted using these doses, as these doses and thus risks might have been slightly lower if these substructures were included in the optimiser. While our cohort received contemporary RT, there was scope to further optimise treatment e.g. reduce PTV margins for patients undergoing DIBH, add further OAR dose volume constraints. Despite these limitations, the doses presented and thus the estimated risks will likely be of interest to the wider lymphoma community and are reflective of a ‘real world’ cohort as many centres worldwide are currently introducing DIBH and advanced VMAT. This study includes patients treated 2016–2019 prior to updated recommendations of dose constraints published by the International Lymphoma Radiation Oncology Group in 2020 [4]. We have since revised our OAR dose constraints in line with this guideline and our results may influence other radiotherapy centres to also update their OAR dose constraints. Another limitation is that excess risks from smoking in the Irish population were not included in the risk prediction due to lack of separate population rates for smokers and never-smokers in Ireland. Smoking increases the risk of CVD and lung cancer and therefore we might have underestimated the risks for some patients. Smoking may increase the background AMR_30_ (and thus excess treatment-related risk) from CVD and lung cancer up to 14-fold compared to never smokers [11]. Of note, incidence of CVD and second cancers were not calculated in this study. Incidence of CVD and breast cancer in particular would be higher than mortality risk, with additional effects on survivors’ quality of life and impact on health services research, therefore surveillance is even more crucial in these patients. Incidence rates in Ireland were not available for the cardiac endpoints reported.

## Conclusions

In conclusion, for patients with mediastinal lymphoma predicted excess mortality risks from CVD and second cancers remain clinically significant despite contemporary chemotherapy and photon RT. Ongoing efforts to reduce the toxicity of combined modality treatment and implement techniques such as DIBH, reduced margins and advanced radiotherapy techniques including PBT are strongly encouraged.

## Supplementary Information

Below is the link to the electronic supplementary material.

Supplementary file1 (DOC 46 KB)

## Data Availability

Research data are available from the corresponding author on reasonable request.

## References

[1] André MPE, Girinsky T, Federico M, Reman O, Fortpied C, Gotti M (2017). Early positron emission tomography response–adapted treatment in stage I and II Hodgkin lymphoma: final results of the randomized EORTC/LYSA/FIL H10 trial. J Clin Oncol.

[2] van Leeuwen FE, Ng AK (2016). Long-term risk of second malignancy and cardiovascular disease after Hodgkin lymphoma treatment. Hematology Am Soc Hematol Educ Program.

[3] Radford J, Illidge T, Counsell N, Hancock B, Pettengell R, Johnson P (2015). Results of a trial of PET-directed therapy for early-stage Hodgkin’s lymphoma. N Engl J Med.

[4] Wirth A, Mikhaeel NG, Aleman BMP, Pinnix CC, Constine LS, Ricardi U (2020). Involved site radiation therapy in adult lymphomas: an overview of international lymphoma radiation oncology group guidelines. Int J Radiat Oncol Biol Phys.

[5] Engert A, Plütschow A, Eich HT, Lohri A, Dörken B, Borchmann P (2010). Reduced treatment intensity in patients with early-stage Hodgkin’s lymphoma. N Engl J Med.

[6] Paumier A, Ghalibafian M, Gilmore J, Beaudre A, Blanchard P, el Nemr M (2012). Dosimetric benefits of intensity-modulated radiotherapy combined with the deep-inspiration breath-hold technique in patients with mediastinal Hodgkin’s lymphoma. Int J Radiat Oncol Biol Phys.

[7] Petersen PM, Aznar MC, Berthelsen AK, Loft A, Schut DA, Maraldo M (2015). Prospective phase II trial of image-guided radiotherapy in Hodgkin lymphoma: benefit of deep inspiration breath-hold. Acta Oncol.

[8] Fiandra C, Filippi AR, Catuzzo P, Botticella A, Ciammella P, Franco P (2012). Different IMRT solutions vs 3D-conformal radiotherapy in early stage Hodgkin’s lymphoma: dosimetric comparison and clinical considerations. Radiat Oncol.

[9] Voong KR, McSpadden K, Pinnix CC, Shihadeh F, Reed V, Salehpour MR (2014). Dosimetric advantages of a “butterfly” technique for intensity-modulated radiation therapy for young female patients with mediastinal Hodgkin’s lymphoma. Radiat Oncol.

[10] Starke A, Bowden J, Lynn R, Hall K, Hudson K, Rato A (2018). Comparison of butterfly volumetric modulated arc therapy to full arc with or without deep inspiration breath hold for the treatment of mediastinal lymphoma. Radiother Oncol.

[11] Ntentas G, Dedeckova K, Andrlik M, Aznar MC, Shakir R, Ramroth J, et al. Proton therapy in supradiaphragmatic lymphoma: predicting treatment-related mortality to help optimize patient selection. Int J Radiat Oncol Biol Phys. 2022;112(4):913–25. 10.1016/j.ijrobp.2021.10.151 PMC8865523 34762970

[12] van Nimwegen FA, Schaapveld M, Janus CP, Krol AD, Petersen EJ, Raemaekers JM (2015). Cardiovascular disease after Hodgkin lymphoma treatment: 40-year disease risk. JAMA Intern Med.

[13] Specht L, Yahalom J, Illidge T, Berthelsen AK, Constine LS, Eich HT (2014). Modern radiation therapy for Hodgkin lymphoma: field and dose guidelines from the International Lymphoma Radiation Oncology Group (ILROG). Int J Radiat Oncol Biol Phys.

[14] Feng M, Moran JM, Koelling T, Chughtai A, Chan JL, Freedman L (2011). Development and validation of a heart atlas to study cardiac exposure to radiation following treatment for breast cancer. Int J Radiat Oncol Biol Phys.

[15] Drake RA, Vogl W, Mitchell A, Tibbitts R, Richardson P (2020). Gray's atlas of anatomy.

[16] IMAIOS. E-anatomy [Internet]. 2021 [cited 2022 Jul 09]. Available from: https://www.imaios.com/en/e-Anatomy.

[17] Duane F, Aznar MC, Bartlett F, Cutter DJ, Darby SC, Jagsi R (2017). A cardiac contouring atlas for radiotherapy. Radiother Oncol.

[18] Houlihan OA, Rangaswamy G, Dunne M, Rohan C, O’Neill L, Chalke S (2021). Deep inspiration breath hold versus free breathing technique in mediastinal radiotherapy for lymphoma. BJR Open.

[19] Cutter DJ, Ramroth J, Diez P, Buckle A, Ntentas G, Popova B (2021). Predicted risks of cardiovascular disease following chemotherapy and radiotherapy in the UK NCRI RAPID trial of positron emission tomography–directed therapy for early-stage Hodgkin lymphoma. J Clin Oncol.

[20] De Bruin ML, Dorresteijn LDA, van’t Veer MB, Krol ADG, van der Pal HJ, Kappelle AC (2009). Increased risk of stroke and transient ischemic attack in 5-year survivors of Hodgkin lymphoma. J Natl Cancer Inst.

[21] van Nimwegen FA, Schaapveld M, Cutter DJ, Janus CP, Krol AD, Hauptmann M (2016). Radiation dose-response relationship for risk of coronary heart disease in survivors of Hodgkin lymphoma. J Clin Oncol.

[22] van Nimwegen FA, Ntentas G, Darby SC, Schaapveld M, Hauptmann M, Lugtenburg PJ (2017). Risk of heart failure in survivors of Hodgkin lymphoma: effects of cardiac exposure to radiation and anthracyclines. Blood.

[23] Cutter DJ, Schaapveld M, Darby SC, Hauptmann M, van Nimwegen FA, Krol ADG, et al. Risk for valvular heart disease after treatment for Hodgkin lymphoma. J Natl Cancer Inst. 2015; 107(4). 10.1093/jnci/djv008 PMC4394894 25713164

[24] Maraldo MV, Lundemann M, Vogelius IR, Specht L (2015). A new method to estimate doses to the normal tissues after past extended and involved field radiotherapy for Hodgkin lymphoma. Radiother Oncol.

[25] Travis LB, Gospodarowicz M, Curtis RE, Clarke EA, Andersson M, Glimelius B (2002). Lung cancer following chemotherapy and radiotherapy for Hodgkin’s disease. J Natl Cancer Inst.

[26] Gilbert ES, Stovall M, Gospodarowicz M, Van Leeuwen FE, Andersson M, Glimelius B (2003). Lung cancer after treatment for Hodgkin's disease: focus on radiation effects. Radiat Res.

[27] Swerdlow AJ, Cooke R, Bates A, Cunningham D, Falk SJ, Gilson D (2012). Breast cancer risk after supradiaphragmatic radiotherapy for Hodgkin’s lymphoma in England and Wales: a national cohort study. J Clin Oncol.

[28] Schaapveld M, Aleman BM, van Eggermond AM, Janus CP, Krol AD, van der Maazen RW (2015). Second cancer risk up to 40 years after treatment for Hodgkin's lymphoma. N Engl J Med.

[29] Lindsay MM, Ethel SG, Marilyn S, EvL Flora, Graça MD, Charles FL (2014). Risk of esophageal cancer following radiotherapy for Hodgkin lymphoma. Haematologica.

[30] Ricardi U, Maraldo MV, Levis M, Parikh RR (2019). Proton therapy for lymphomas: current state of the art. Onco Targets Ther.

[31] Levis M, Filippi AR, Fiandra C, De Luca V, Bartoncini S, Vella D (2019). Inclusion of heart substructures in the optimization process of volumetric modulated arc therapy techniques may reduce the risk of heart disease in Hodgkin’s lymphoma patients. Radiother Oncol.

[32] Dabaja BS, Hoppe BS, Plastaras JP, Newhauser W, Rosolova K, Flampouri S (2018). Proton therapy for adults with mediastinal lymphomas: the International Lymphoma Radiation Oncology Group guidelines. Blood.

[33] Ntentas G, Dedeckova K, Andrlik M, Aznar MC, George B, Kubeš J (2019). Clinical intensity modulated proton therapy for Hodgkin lymphoma: which patients benefit the most?. Pract Radiat Oncol.

[34] Vries Sd, Haaksma ML, Jóźwiak K, Schaapveld M, Hodgson DC, Lugtenburg PJ, et al. Development and validation of risk prediction models for coronary heart disease and heart failure after treatment for Hodgkin lymphoma. J Clin Oncol. 2022; 0(0):JCO.21.02613. 10.1200/JCO.21.02613 35947813

