# Supplementary material for: Predicted cardiac and second cancer risks for patients undergoing VMAT for mediastinal Hodgkin lymphoma
Source: Clin Transl Oncol. 2022 Dec 31;25(5):1368–77. doi: 10.1007/s12094-022-03034-z (PMC10119211; doi:10.1007/s12094-022-03034-z)
Supplement: Supplementary file 1 — Supplementary file1 (DOC 46 KB) [file 12094_2022_3034_MOESM1_ESM.doc]

**Table S1.** Contouring definitions.

| **OAR** |  |  |
| --- | --- | --- |
| Heart | Entire pericardium contoured from just inferior to left pulmonary artery to the most inferior CT slice where pericardium is visible and blends with the diaphragm inferiorly. |  |
| Breasts | Superior border: Upper border of palpable/visible breast tissue |  |
|  | Anterior border: Skin surface |  |
|  | Posterior border: Major pectoral muscle or costae and intercostal muscles |  |
|  | Medial border: Lateral to the medial perforating mammarian vessels; maximally to sternal edge |  |
|  | Lateral border: Lateral breast fold; anterior to lateral thoracic artery |  |
|  |  |  |
|  | Inferior border: At the caudal border of visible breast tissue |  |
| Oesophagus | All mucosa, submucosa and muscular layers contoured from the inferior level of the cricoid cartilage to the gastro oesophageal junction. |  |
| Common carotid arteries | Entire circumference of the common carotid arteries contoured from their bifurcation into the internal and external carotid arteries to the junction with the aorta. |  |
| Left ventricle | The LV was identified anteriorly and to the left of the left atrium, following segmentation of the heart into its four chambers by the septal and atrioventricular groove planes. The septal plane was marked with a straight line through the fat space between the right and left atria. The atrioventricular groove plane lies perpendicular to the septal plane. |  |
| Lungs | Generated by automatic contouring in ARIA Oncology Information System and manually edited to include only visible lung tissue. |  |
| Valves | Generated as per cardiac atlas described by Feng et al. (14) |  |

**Table S2:** Percentage of patients with mediastinal Hodgkin lymphoma that may benefit from proton beam therapy.

| ***Longitudinal overlap CTV/Heart*** | **n** | **(%)** |
| --- | --- | --- |
| <40% overlap | 26 | (59) |
| ≥40% overlap | 18 | (41) |
| ***CTV inferior extension versus ﻿left main stem coronary artery*** |  |  |
| At and above | 13 | (30) |
| Below | 31 | (70) |
| ***CTV inferior extension versus vertebral thoracic level*** |  |  |
| At and above 7^th^ thoracic level | 21 | (48) |
| Below 7^th^ thoracic level | 23 | (52) |
| ***Axilla irradiated*** |  |  |
| Yes | 16* | (36) |
| No | 28 | (64) |

*10 female patients
